# Effect of environmental exposome and influenza infection on febrile seizure in children over 22 years: a time series analysis

**DOI:** 10.1007/s00484-024-02711-8

**Published:** 2024-05-31

**Authors:** Xiaoting Jiang, Conglu Li, Qianying Yue, Yuchen Wei, Yawen Wang, Xiang Qian Lao, Guozhang Lin, Ka Chun Chong

**Affiliations:** 1 Jockey Club School of Public Health and Primary Care, The Chinese University of Hong Kong, Hong Kong Special Administrative Region, Prince of Wales Hospital, Shatin, New Territories China; 2 Centre for Health Systems and Policy Research, The Chinese University of Hong Kong, Shenzhen, Hong Kong Special Administrative Region China; 3 Department of Biomedical Sciences, City University of Hong Kong, Kowloon, Hong Kong Special Administrative Region China; 4 Clinical Trials and Biostatistics Laboratory, Shenzhen Research Institute, The Chinese University of Hong Kong, Shenzhen, China

**Keywords:** Febrile seizure, Seasonal influenza, Ambient temperature, Time-series, Distributed-lag non-linear model, Subtropics

## Abstract

Febrile seizures are convulsions predominately occurring in young children. The effects of various exposomes, including influenza infection and external environmental factors, on febrile seizures have not been well-studied. In this study, we elucidated the relationships between ambient temperature, air pollutants, influenza infection, and febrile seizures using 22-year territory-wide hospitalization data in Hong Kong. The aggregated data were matched with the meteorological records and air pollutant concentrations. All-type and type-specific influenza-like illness positive (ILI+) rates were used as proxies for influenza activity. Distributed lag non-linear model in conjunction with the quasi-poisson generalized additive model was used to examine the associations of interest. According to the results, all-type influenza infections were significantly associated with an increased risk of hospital admissions for febrile seizures (cumulative adjusted relative risk [ARR] = 1.59 at 95th percentile vs. 0; 95% CI, 1.51–1.68). The effect of ILI + A/H3N2 on febrile seizure was more pronounced than other type-specific ILI + rates. A low mean ambient temperature was identified as a significant risk factor for febrile seizures (cumulative ARR = 1.50 at 5th percentile vs. median; 95% CI, 1.35–1.66), while the redox-weighted oxidant capacity and sulfur dioxide were not associated with febrile seizures. In conclusion, our study underscores that influenza infections and exposure to cold conditions were related to an increased risk of febrile seizures in children. Thus, we advocate for influenza vaccination before the onset of the cold season for children to mitigate the burden of febrile seizures.

**Supplementary Information:**

The online version contains supplementary material available at 10.1007/s00484-024-02711-8.

## Introduction

Febrile seizures, also known as febrile convulsions or fever fits, refers to convulsions that is associated with fevers but without the presence of intracranial infection, hypoglycemia, or an acute electrolyte imbalance (Sadleir and Scheffer 2007). Febrile seizures mainly occur in infants and children aged six months to six years, affecting 2–5% of those under five, with even higher rates observed in developing settings (Verity et al. 1985). The majority of these incidents are seen in children under the age of 3 years, primarily because their brains are more susceptible due to immaturity (Sharawat et al. 2016). Symptoms of febrile seizure manifestation typically include fever, lose consciousness, irregular breathing, arms and legs twitching, eyes rolling upwards (Paul et al. 2015). While febrile seizures are generally benign and not indicative of serious health issues in children (Nelson and Ellenberg 1976), they can give rise to unease or even extreme fear for parents (Baumer et al. 1981).

Febrile seizures are triggered by fever, which can result from any illness that elevates body temperature. Among these, viral infection stands as a prevalent cause of fever leading to febrile seizures (Smith et al. 2019). An observational study by Francis et al. (2016) highlighted that 71% of the febrile seizure cases were associated with viral infections, while Lewis et al. (1979) reported an even higher correlation, with 86% of cases being virus-related. Specifically, influenza was recognized as a significant viral contributor to febrile seizures. A five-year retrospective study (Chung and Wong 2007) showed that among five common viruses that infect human beings, including influenza, adenovirus, parainfluenza, respiratory syncytial virus and rotavirus, influenza emerged as the most frequently associated virus (17.6%) in febrile seizure incidence among children. Moreover, this study noted that 20.8% of children hospitalized with influenza exhibited febrile seizures, a higher relative risk compared to other infections. Supporting this, a retrospective cohort study conducted by Chiu et al. (2001) found that influenza A was implicated in 19.5% of febrile seizure cases, a rate significantly higher than whose associated with parainfluenza and adenovirus.

Recent findings have highlighted the environmental exposome, including air pollutants and meteorological factors, as significant risk factors for febrile seizures. Christensen et al. (2022) observed a notably higher occurrence of febrile seizure during winter compared to the summer, echoing another study conducted in South Korea by Kin et al.(Kim et al. 2017) Analyses from two time-series studies indicated that lower ambient mean temperature, higher O_3_ concentration and lower NO_2_ concentration were statistically significantly associated with hospital visit due to febrile seizure (Kim et al. 2017, 2019). However, the findings were inconsistent with the other investigations (Hjortebjerg et al. 2018; Kawakami et al. 2020), indicating a need for further research to fully understand the environmental influences on febrile seizure risk.

To the best of our knowledge, only one study conducted in Japan (Kawakami et al. 2020) has quantified the impact of multiple exposome, including influenza infection and environmental factors on febrile seizure simultaneously, though the potential lagged effect of the factors has not been accounted for in this research (Kim et al. 2017; Chai et al. 2020). In this study, we aim to elucidate the relationship between cold conditions, air pollutants, influenza infections, and febrile seizure manifestations, leveraging comprehensive population-based data in Hong Kong.

## Materials and methods

### Data source

The study period is from June 1998, to December 2019. Weekly hospital admissions within the public healthcare sector, diagnosed with febrile seizure (International Classification of Diseases ICD9: 780.31) and weekly all-cause hospital admissions were obtained from the surveillance data provided by the Hong Kong Hospital Authority. Children aged 0 to 3 years old who had been diagnosed with febrile seizure were included in the study. Weekly consultation rates for influenza-like illness (ILI), defined as a fever of greater than or equal to 38.5^o^C, in addition to cough and sore throat, were collected from the Centre for Health Protection, Hong Kong (Cowling et al. 2006). Weekly influenza activity was approximated by influenza-like illness positive (ILI+) rate in this study (Chong et al. 2022; Li et al. 2023; Xiong et al. 2023). ILI + rate was derived by multiplying the proportion of community ILI consultations and the proportion of positive respiratory specimens for a particular influenza subtype, utilizing the surveillance data from the Centre for Health Protection, Hong Kong. The weekly combined rate of all influenza strains (i.e., ILI + total) and rates for specific influenza strains, including ILI + A/H1N1, A/H3N2 and B, were derived for this analysis. Weekly mean ambient temperature data were obtained from the Hong Kong Observatory. Weekly air pollutant data, including the mean concentration of NO_2_, SO_2_, O_3_ and PM_2.5_, were derived by averaging the data collected from over 10 monitoring stations of the Environmental Protection Department in Hong Kong. To ensure the alignment of data, all datasets, including ILI and environmental variables, were consistently compiled on a weekly basis, from Sunday to Saturday.

### Statistical analysis

Previous studies have demonstrated that both influenza and environmental factors could exert delayed and non-linear effects on health outcomes (Kim et al. 2019; Mohammad et al. 2021). In order to examine the associations of interest while accounting for the abovementioned effects, we employed a quasi-poisson generalized additive model in combination with the distributed lag non-linear model (DLNM) (Gasparrini et al. 2010). The weekly number of hospital admissions due to febrile seizures was set as the outcome variable. The model used for the analysis is as follows:

$$ \log ({\mu _t})\, = \,intercept\, + \,cb(lLl\,{ + _t};lag)\, + $$

$$ \,cb(tem{p_t};lag) + \,cb({O_{xt}};\,lag)\, $$

$$ + \,cb(S{O_{2t}};\,lag) + s(yea{r_t}) + $$

$$ (yea{r_t}) + s(wee{k_t}) + offse{t_t} $$

where *µ*_*t*_ is the expected number of hospital admissions for febrile seizure in week t. *cb(.)* represents a cross-basis function that models the common exposure-response relationship and lagged effects of explanatory variables at once (Gasparrini et al. 2011). *s(.)* denotes a smoothing spline function. *ILI +* _*t*_ represents the rate of ILI + in week *t*. Mean temperature (*temp*_*t*_) in week *t* was included to explore the meteorological effects on the outcome. The weekly mean concentration of sulfur dioxide (*SO*_*2t*_) and redox-weighted oxidant capacity (*O*_*xt*_) were involved to explore the effects of air pollutants. O_x_ represents the combined oxidant capacity of O_3_ and NO_2_, and it was employed to avoid colinearity between O_3_ and NO_2_. O_x_ is the weighted average of O_3_ and NO_2_ by their respective redox potentials (i.e. O_x_ = (1/3)NO_2_+ (2/3)O_3_) (Weichenthal et al. 2017), and it has been demonstrated as a better indicator of atmospheric oxidative capacity than unweighted oxidant capacity (Guo et al. 2018). The effect of PM_2.5_ was examined separately from O_x_ and SO_2_ to avoid a colinearity. *year*_*t*_ and *week*_*t*_ denote year and week of the year, and they were included to account for the long-term trends and seasonality, respectively. *offset*_*t*_ is the offset term of this regression model, which is the natural logarithm of the number of all-cause hospital admissions of children aged 0 to 3 in week t. It serves to adjust our analysis for differences in the population at risk each week, allowing the comparison of febrile seizure rates across different covariates. The degrees of freedom of the exposure variables and the lag effect were chosen in a range of two to five and optimized by minimizing the generalized cross-validation score. The maximum lag time was set to 2 weeks following the similar studies (Chong et al. 2022; Mohammad et al. 2021). The impact of influenza and environmental exposures on febrile seizure admissions was quantified by cumulative adjusted relative risk (ARR), which accumulates the relative risk over the lag period, along with the corresponding 95% confidence intervals (CI). An ARR with 95% CI excluding one indicates statistically significance. The reference level of ILI + rates was set as zero. The reference values for ambient temperature were set to its median, and the reference values for air pollutants were set to their respective 5th percentiles (Leung et al. 2021; Mohammad et al. 2021). Adjusted R-square was used to evaluate the goodness of fit of our models, with higher values indicating a better fit.

### Sensitivity analysis

Given the unique health landscape during the 2009 influenza pandemic in Hong Kong, which potentially altered the seasonality of hospital admission trends and could affect the robustness and generalizability of our study outcomes, we conducted a sensitivity analysis by excluding the data from 2009. This exclusion aimed to evaluate the pandemic’s impact on the observed magnitude of febrile seizures in relation to total and type-specific ILI + rates and environmental factors. Further, with the Environmental Protection Department of the government of Hong Kong launching the Air Quality Health Index (AQHI) on December 30, 2013, to provide the public with real-time air pollutant data with relevant health advice (Environmental Protection and Department 2013), we hypothesized that public behavior and exposure to environmental pollutants might have changed significantly thereafter. Hence, a second sensitivity analysis was conducted by excluding the data collected after week 52, 2013 to assess the potential influence of these changes on our study outcomes. For both sensitivity analyses, we replicated the initial statistical procedures on the adjusted datasets to compare the outcomes with and without the excluded periods. Differences in results between the initial analysis and each sensitivity analysis were carefully examined to assess the impact of these exclusions on our findings. This comparative approach was designed to specifically ascertain the stability of the relationship between ILI+, environmental factors, and the incidence of febrile seizures under varying conditions.

All statistical analyses were conducted using the “dlnm” and “mgcv” packages in the R environment (version 4.1.2).

### Ethics approval and consent to participate

Ethics approval and consent to participate are not required as only retrospective aggregated data were used.

## Results

**Fig. 1:**
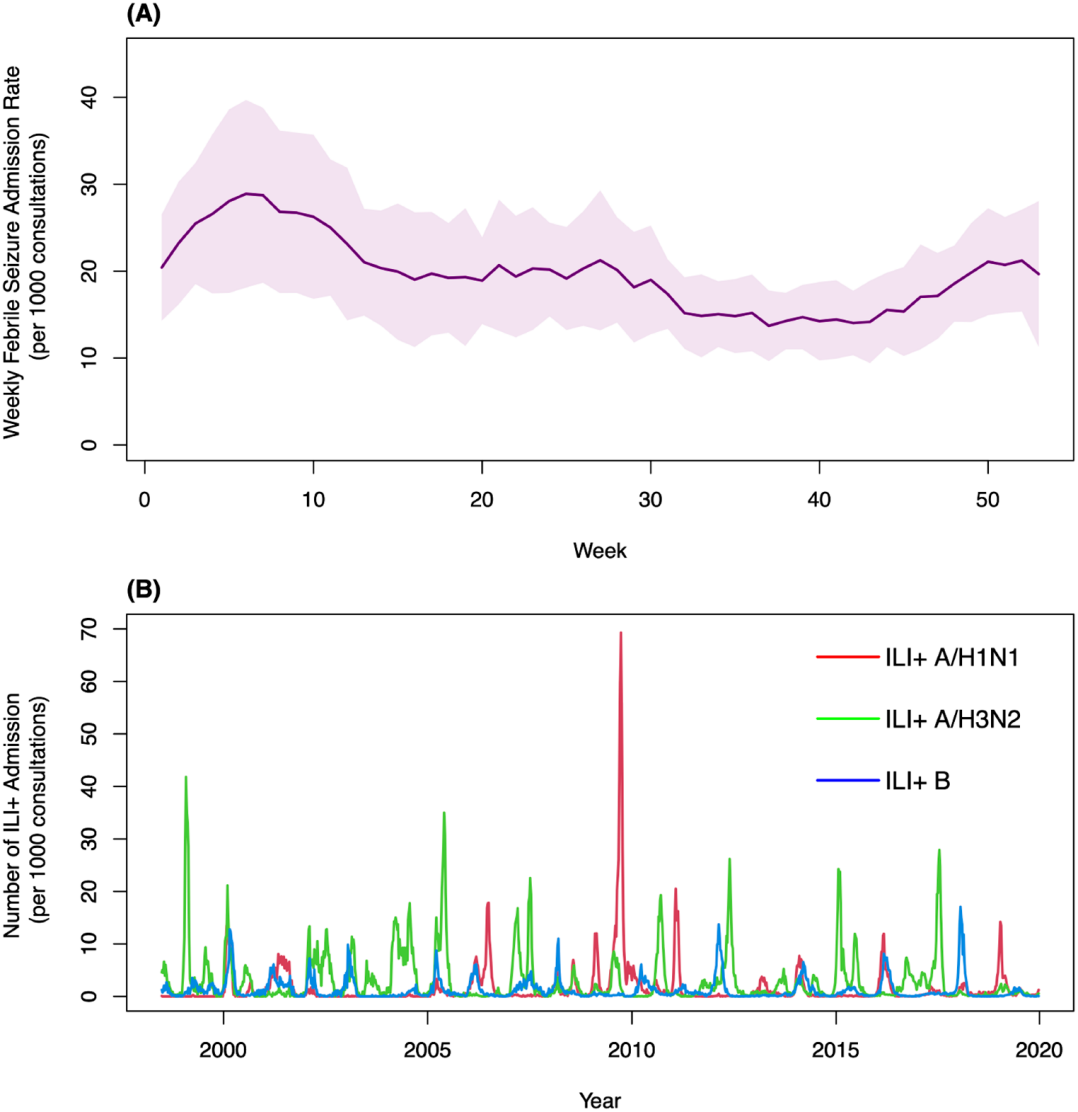
Seasonality of (**A**) admission rate for febrile seizure for children aged 0 to 3 years old from 1998 to 2019, and (**B**) influenza-like illness + rate from 1998 to 2019. The admission rates due to febrile seizure are expressed as mean ± one standard deviation by week

**Fig. 2:**
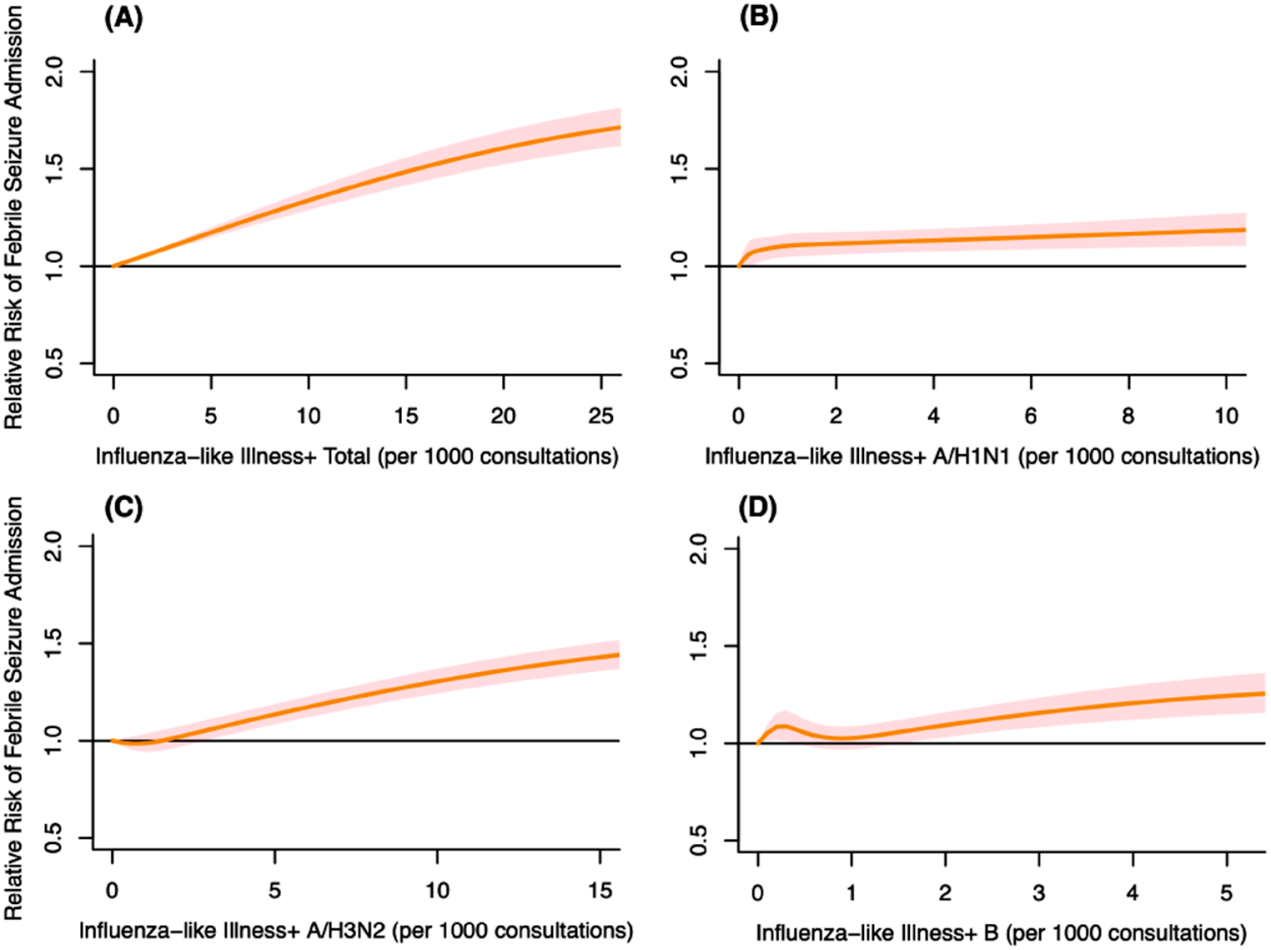
Cumulative adjusted relative risks (ARRs) with 95% confidence interval on febrile seizure admissions for children aged 0 to 3 years old against different influenza-like illness (ILI) + rates. (A) ILI+ total; (B) ILI+ A/H1N1; (C) ILI+ A/H3N2; (D) ILI+ B.

From June 1998 to December 2019, we recorded a total of 41,407 hospital admissions for febrile seizures among children aged 0 to 3 years. The seasonality of febrile seizure rate is depicted in Fig. 1. The weekly median (inter-quartile range) rate of febrile seizures was 18 (14–24) per 1000 consultations, and the weekly median rates of ILI + total, ILI + A/H1N1, ILI + A/H3N2, and ILI + B were 3.32 (1.19–7.76), 0.22 (0.01–1.11), 0.93 (0.26–3.31), and 0.47 (0.15–1.43) per 1000 consultations respectively (Table 1). In 2009, Hong Kong experienced an aberrant upsurge in ILI + A/H1N1 rate (Fig. 2). The weekly median levels of temperature, was 24.6^o^C (19.5^o^C -28.0^o^C). The weekly O_3_ level was 37.1 µg/m^3^ (26.2 µg/m^3^-49.4 µg/m^3^), and the weekly SO_2_ and NO_2_ levels were13.3 µg/m^3^ (9.2 µg/m^3^-18.7 µg/m^3^) and 51.5 µg/m^3^ (41.5 µg/m^3^-60.5 µg/m^3^), respectively. The weekly PM_2.5_ level was 30.3 µg/m^3^ (20.6 µg/m^3^-42.8 µg/m^3^). Kendall’s Tau correlation coefficients between environmental variables are shown in Table S1 in the Appendix. NO_2_, O_3_ and PM_2.5_ have moderate to strong correlations with one another. The adjusted R-square for the models ranged from 0.524 to 0.668 (Table S2, Appendix), indicating moderate to good fitting of the models.

**Table 1:** Descriptive statistics of weekly total number of hospital admission rates for children aged 0 to 3 years old due to febrile seizure, weekly influenza-like illness (ILI) + rates, meteorological variables, and air pollutants over 1998–2019 in Hong Kong

		5th percentile	25th percentile	Median	75th percentile	95th percentile
Weekly FS admission rates for children aged 0 to 3 years old (per 1000 admissions)		10	14	18	24	34
Weekly ILI + rates (per 1000 consultations)	ILI + total	0.38	1.19	3.32	7.76	19.24
	ILI + A/H1N1	0.00	0.01	0.22	1.11	7.07
	ILI + A/H3N2	0.00	0.26	0.93	3.31	12.68
	ILI + B	0.00	0.15	0.47	1.43	5.14
Environmental covariates	Mean temperature (^◦^C)	15.11	19.52	24.61	28.00	29.69
	O_3_ (*µ*g/m^3^)	22.83	32.92	43.00	53.27	66.02
	SO_2_ (*µ*g/m^3^)	5.61	9.15	13.31	18.69	27.86
	NO_2_ (*µ*g/m^3^)	30.76	41.49	51.50	60.46	76.99
	PM_2.5_(*µ*g/m^3^)	10.93	20.55	30.30	42.78	62.24

FS: febrile seizure; ILI: influenza-like illness; O_3_: ozone; SO_2_: sulfur dioxide; NO_2_: nitrogen dioxide; PM_2.5_: fine particulate matter

The effects of ILI + total and type-specific ILI + on febrile seizures are shown in Fig. 2 while the lag-specific effects are presented in Table 2. The cumulative ARR of febrile seizures increased to 1.59 (95% CI, 1.51–1.68) when the ILI + total rate reached the 95th percentile. Similar monotonic increasing trends of the risks were observed for type-specific ILI + rates, with the effect of ILI + A/H3N2 on febrile seizure being more pronounced. The cumulative ARR of febrile seizure rose to 1.38 (95% CI, 1.31–1.45) when the ILI + ILI + A/H3N2 rate was at the 95th percentile. The most substantial risk for febrile seizure admissions due to all ILI + was identified at week zero, with all risks remaining significantly above 1 at a one-week lag.

**Table 2:** Adjusted relative risk (95% confidence intervals) of ILI + rates at different lag times on hospital admissions due to febrile seizure when the influenza-like illness (ILI) + rate increases to 95th percentile

	ILI + total	ILI + A/H1N1	ILI + A/H3N2	ILI + B
*Lag zero*	**1.69 (1.56–1.82)**	**1.12 (1.02–1.24)**	**1.45 (1.31–1.60)**	**1.17 (1.04–1.31)**
*Lag at first week*	**1.17 ( 1.15–1.19)**	**1.05 (1.03–1.07)**	**1.11 (1.09–1.13)**	**1.08 (1.05–1.11)**
*Lag at second week*	0.81 (0.75–0.87)	0.98 (0.89–1.09)	0.86 (0.77–0.95)	0.99 (0.88–1.21)
*Overall*	**1.59 (1.51–1.68)**	**1.16 (1.09–1.23)**	**1.38 (1.31–1.45)**	**1.25 (1.15–1.35)**

ILI: influenza-like illness; **Bold** indicates a statistical significance. The reference level of ILI + rates was set as zero

**Fig. 3:**
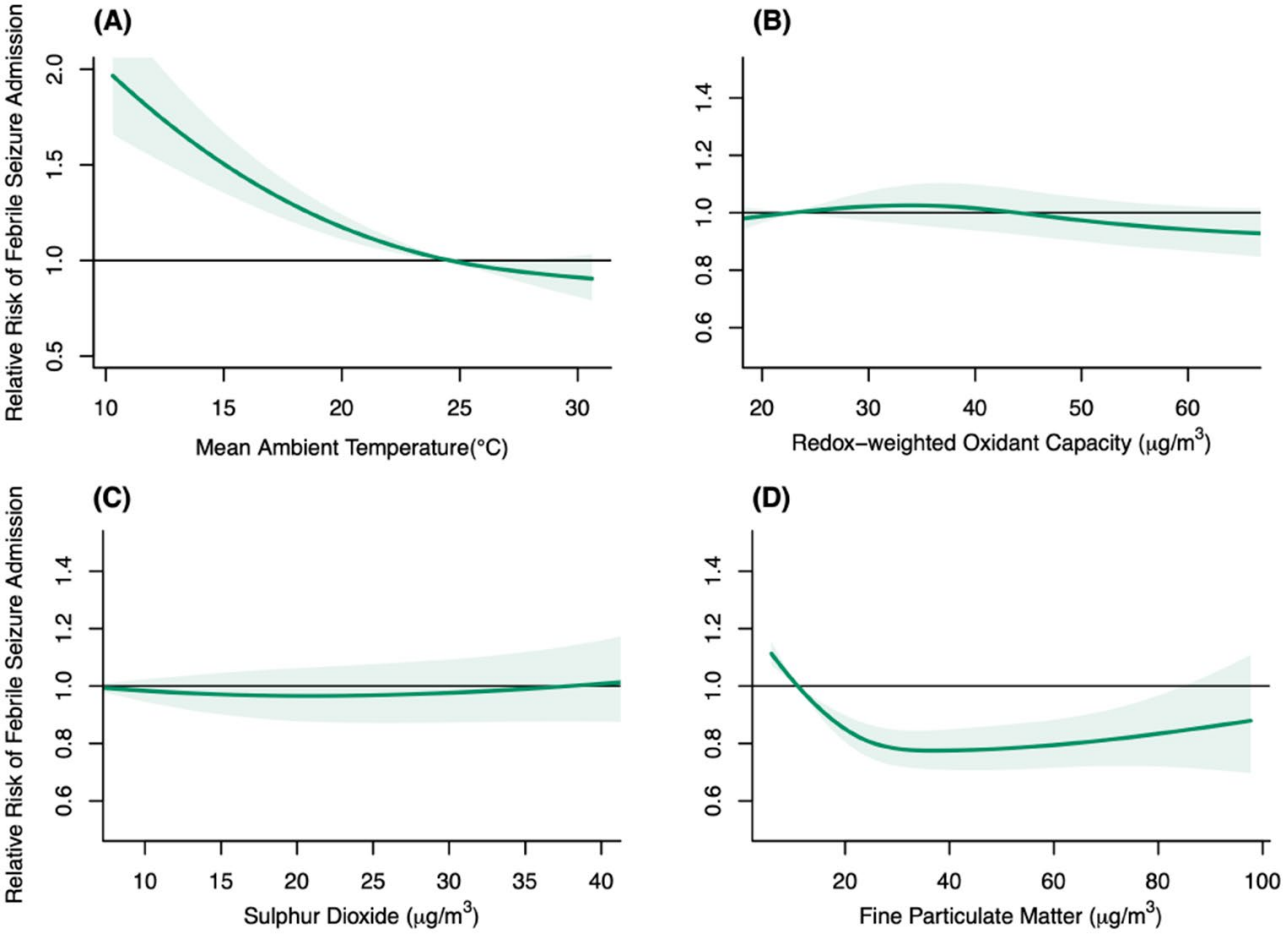
Cumulative adjusted relative risks (ARRs) with 95% confidence interval on febrile seizure admissions for children aged 0 to 3 years old against ambient temperature and pollutant covariates. (A) Mean ambient temperature; (B): Redox-weighted oxidant capacity; (C) Sulfur dioxide; (D) Fine particulate matter.

A low ambient temperature was statistically significantly associated with a higher risk of febrile seizures (Fig. 3). Using the median temperature (24.6ºC) as the reference value, the cumulative ARRs of febrile seizure admissions at the 5th percentile (i.e., 15.1^o^C) was 1.50 (95% CI: 1.35–1.66). The cumulative ARRs of PM_2.5_ were statistically significantly below unity over the 5th to the 99th concentration level, with the cumulative ARRs at the 95th percentile (65.3 µg/m^3^) being 0.80 (95% CI: 0.72–0.89). The cumulative ARRs for O_x_ and SO_2_ were not significant over the entire observed range.

In the sensitivity analysis, we meticulously analyzed the differences between the outcomes of the initial analysis and each subsequent sensitivity analysis. A comparative review of Figure S1 against Figs. 1 and 2 revealed a general consistency in the effects of both total and type-specific ILI+, as well as all environmental factors, on febrile seizures (Figure S1, Appendix). This indicates that the relationships identified in our primary analysis are largely unaffected by the exclusion of data from 2009. Similarly, our results also remained robust upon excluding data collected after the AQHI was introduced, underscoring the stability of our results across various conditions (Figure S2, Appendix).

## Discussion

In this study, we elucidated the relationships between the external exposome (i.e., ambient temperature and air pollutants), the internal exposome (influenza infection), and febrile seizures using territory-wide hospitalization data from Hong Kong. We identified seasonal influenza as a risk factor for febrile seizures, consistent with previous observational studies (Chung and Wong 2007; Chiu et al. 2001) that highlighted the current understanding of the pathogenic mechanisms of febrile seizures (Mosili et al. 2020). Influenza infection can induce an inflammatory response and then elevate the levels of cytokines such as interleukin-1β, IL-6, and tumour necrosis factor-α (Gu et al. 2021; Han and Han 2023; Vezzani and Viviani 2015). The increase in these cytokines can subsequently raise core body temperature, further triggering convulsions. In Addition, we demonstrated that the associations between febrile seizures and influenza A/H1N1, A/H3N2 and B were all statistically significant at the population level. Among the influenza types, the effect of influenza A/H3N2 on febrile seizures was more pronounced, aligning with the findings of other observational studies. These studies reported that influenza A/H3N2 is associated with more severe clinical symptoms than influenza A/H1N1 and B, including a higher frequency of fever and higher body temperature (Kaji et al. 2003; Silvennoinen et al. 2015). Considering the safety and protective effect of pediatric influenza vaccination (France et al. 2004; Jefferson et al. 2018), we recommend seasonal vaccination for vulnerable children to protect them from influenza infection and febrile seizures.

Our study found that cold conditions were independently associated with a higher risk of febrile seizure admissions, regardless of influenza activity, being in line with previous research (Kim et al. 2017). We speculate that this association could be resulted from the abnormalities of thermoregulation. Exposure to cold ambient temperatures can trigger the hypothalamus to signal the skin, muscles and various organs to produce additional heat to maintain normal body temperature (Osilla et al. 2022). However, since the hypothalamus of infants and children is not fully developed, this process can lead to overproduction and fever. Additionally, some studies indicated that cold temperatures can could weaken the immune response (Hu et al. 2016; Kelley 1984), making people being more susceptible to fever-inducing agents. Along with climate change, it is expected that more extremely cold weathers could occur (Cohen et al. 2020, 2021), the susceptibility of children to febrile seizure is expected to increase.

Regarding the effect of air pollutants, our findings indicated that O_x_ and SO_2_ were not associated with febrile seizure manifestation. PM_2.5_ was found to be a protective factor for febrile seizures in our study. However, the association seemed biologically implausible, given that PM_2.5_ is generally considered harmful to human health, and exposure to PM_2.5_ has been found to induce in systemic inflammation in humans (Schneider et al. 2011; Zhao, 2013). It is possible that various air quality control measures and policies enacted during our study period may explain this unexpected result. Such initiatives likely reduced the concentration of PM_2.5_ (Lin et al. 2018), and may lead to behavioral changes among parents, such as limiting outdoor activities for their children, keeping windows closed to maintain clean indoor air, and using air purifiers. In this case, despite the high ambient levels of PM_2.5_, actual exposure may have been minimized. Nevertheless, to the best of our knowledge, no study has yet delineated the specific biological mechanism by which air pollutants influence febrile seizure or other convulsions, underscoring the need for further experimental investigation.

A major advantage of this study is the data completeness, a feature often missing in research from developing settings (Waruiru et al. 2004). Our analysis utilizes daily data spanning 22 years, emcompassing the majority of hospital admission cases in Hong Kong. Also, the clear and consistent timing and intervals of the data points in the surveillance data allowed for the effective application of DLNM model to account for the lag effects of the variables under study, thereby minimizing bias. Moreover, employing ILI + as a proxy of influenza cases addresses the common issue of under-reporting, enabling more accurate estimations (Mohammad et al. 2020). Given the current uncertainty surrounding the precise mechanisms of febrile seizures, the associations observed between environmental factors and febrile seizure in this study could also offer valuable insights into understanding the underlying biological mechanisms.

This study is subjected to several limitations. Firstly, ecological fallacy may be a problem since we used aggregated data to estimate the associations of interest. Secondly, individual risk factors for febrile seizure manifestation, such as age, genetic predisposition, vaccination and medical history (Chung 2014; Graves et al. 2012; Principi and Esposito 2013), were unable to be accounted for in this study. Thirdly, our study did not account for the effects of other pathogens known to induce febrile seizures, such as respiratory syncytial virus (Leung et al. 2021), due to the unavailability of relevant surveillance data.

## Conclusion

Influenza infections and cold conditions were significant exposomes related to an increased risk of febrile seizures in children. In settings where inpatient resources for managing febrile seizures are limited, it is highly recommended that children of eligible age receive the influenza vaccination to mitigate the incidence of febrile seizures. Additionally, it is crucial to ensure young children are kept warm during cold weather, especially in light of the increase of extreme cold days along with climate change.

## Electronic supplementary material

Below is the link to the electronic supplementary material.

Supplementary Material 1

## Data Availability

The sharing of data is restricted by Hong Kong Hospital Authority. All code used for the analyses can be provided upon request.
