# Supplementary material for: Effect of environmental exposome and influenza infection on febrile seizure in children over 22 years: a time series analysis
Source: Int J Biometeorol. 2024 May 31;68(9):1847–55. doi: 10.1007/s00484-024-02711-8 (PMC11461585; doi:10.1007/s00484-024-02711-8)
Supplement: Supplementary file 1 — Supplementary Material 1 [file 484_2024_2711_MOESM1_ESM.docx]

**Table S1. Kendall's Tau correlation coefficients between air pollutants**

|  | NO_2_ | SO_2_ | O_3_ | PM_2.5_ |
| --- | --- | --- | --- | --- |
| NO_2_ |  |  |  |  |
| SO_2_ | 0.32 |  |  |  |
| O_3_ | 0.26 | -0.14 |  |  |
| PM_2.5_ | 0.64 | 0.37 | 0.31 |  |

**Table S2: Adjusted R^2^ for analyzed models**

|  | Covariates | Adjusted R^2^ |
| --- | --- | --- |
| Model 1 | ILI+ Total, Oxidants, SO_2_, Ambient Temperature, Year, Week, Offset term | 0.663 |
| Model 2 | ILI+ Total, PM2.5, Ambient Temperature, Year, Week, Offset term | 0.668 |
| Model 3 | H1N1, Oxidants, SO_2_, Ambient Temperature, Year, Week, Offset term | 0.539 |
| Model 4 | ILI+ H3N2, Oxidants, SO_2_, Ambient Temperature, Year, Week, Offset term | 0.615 |
| Model 5 | ILI+ B, Oxidants, SO_2_, Ambient Temperature, Year, Week, Offset term | 0.524 |

**
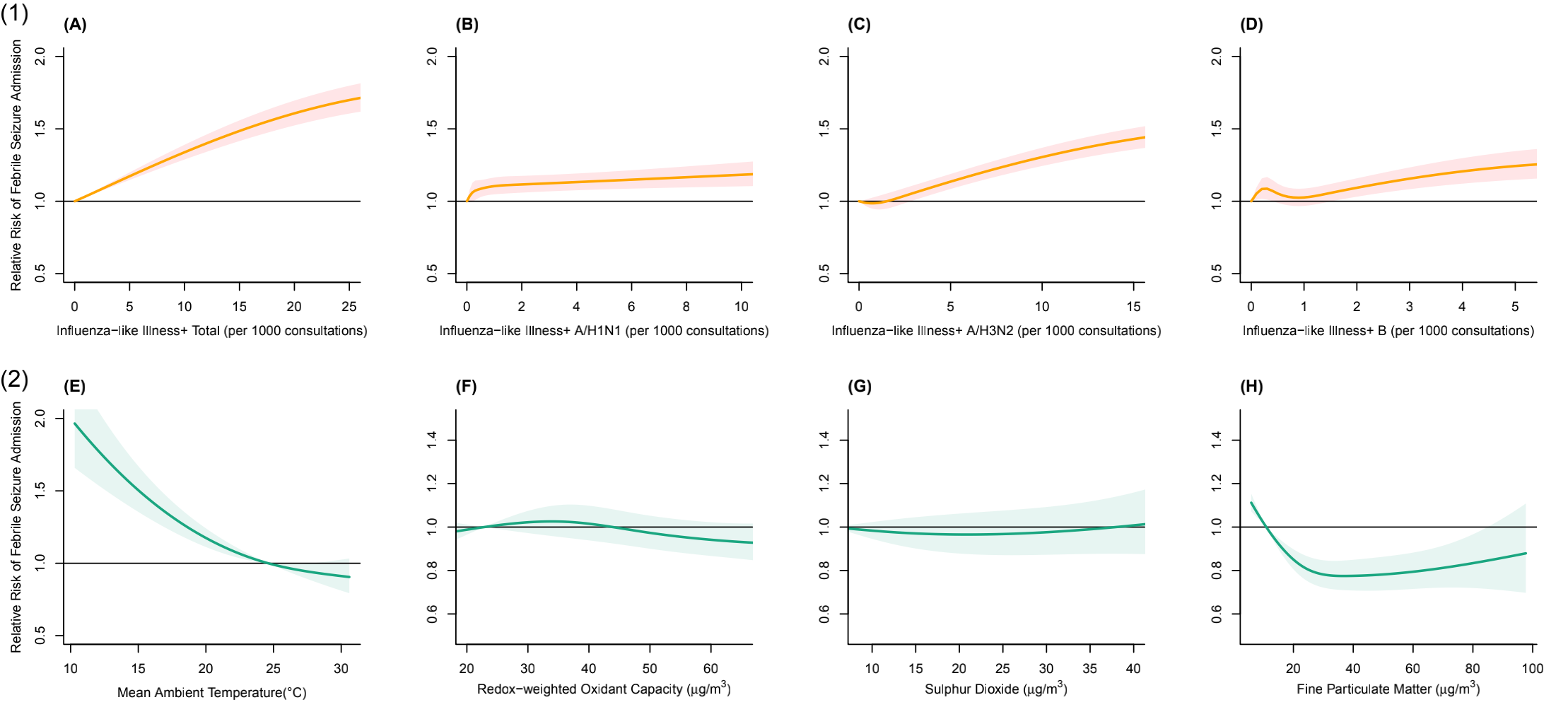
Figure S1. Cumulative adjusted relative risks (ARRs) with 95% confidence interval on febrile seizure admissions for children aged 0 to 3 years old against (1) different influenza-like illness (ILI) + rates and (2) different environmental covariates when the data from 2009 were excluded.**

**
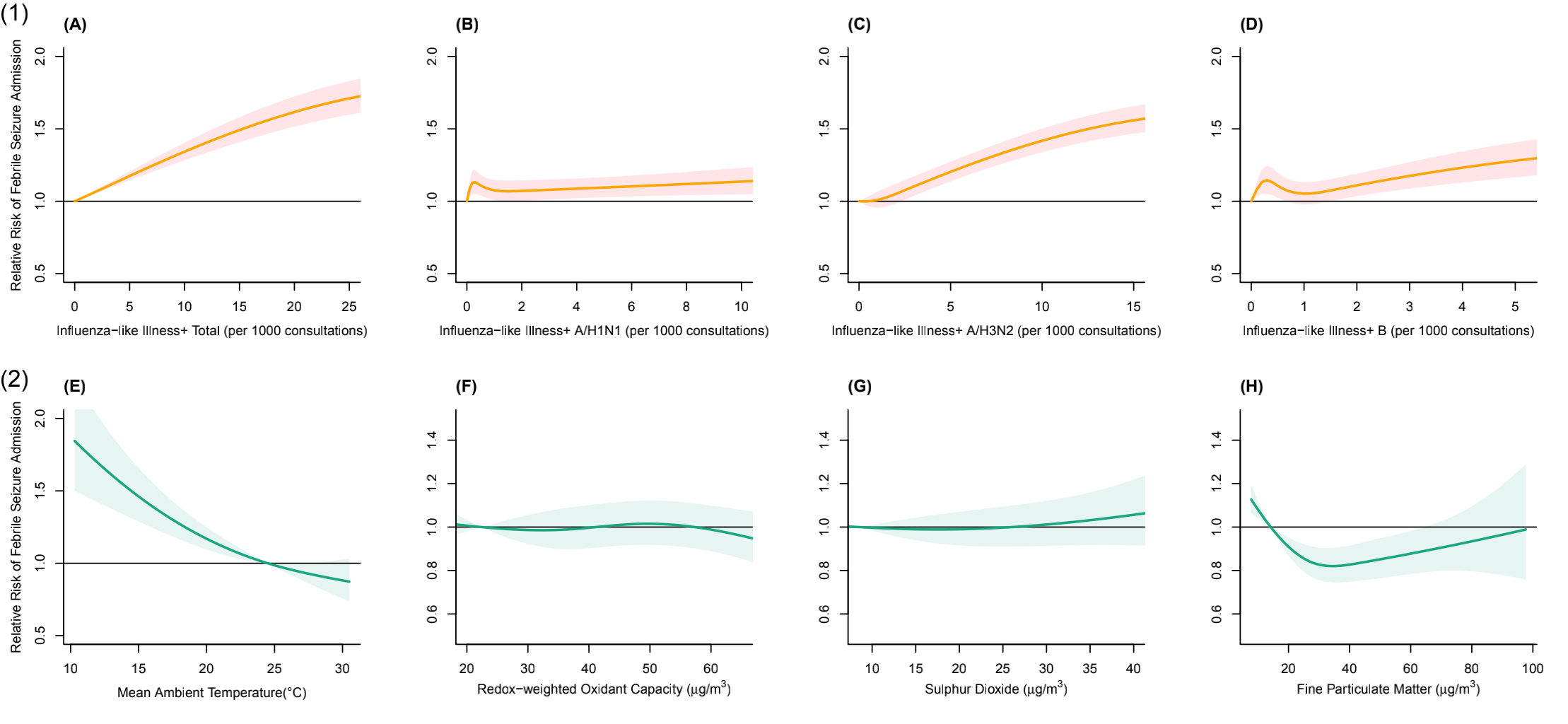
Figure S2. Cumulative adjusted relative risks (ARRs) with 95% confidence interval on febrile seizure admissions for children aged 0 to 3 years old against (1) different influenza-like illness (ILI) + rates and (2) different environmental covariates when the data after Dec 31, 2013 were excluded.**
